# Lifestyle Adjustments in Diabetic Individuals Following Lower Extremity Amputations: A Qualitative Study

**DOI:** 10.7759/cureus.76188

**Published:** 2024-12-22

**Authors:** Ba-Etilayoo Atinga, Albert Henyo, Graham Addah, Christiana A Ayamga, Tulukuu Perekuu

**Affiliations:** 1 Anatomy and Nursing, University of Energy and Natural Resources, Sunyani, GHA; 2 Nursing, University of Energy and Natural Resources, Sunyani, GHA; 3 Paediatrics and Child Health, Sunyani Municipal Hospital, Sunyani, GHA; 4 Nursing, Holy Family Nursing and Midwifery Training College, Berekum, GHA

**Keywords:** amputation, diabetic patients, ghana, lifestyle changes, lower extremities

## Abstract

Background: Diabetes mellitus (DM) often leads to lower extremity amputations when poorly managed. Managing DM in Ghana is difficult due to limited access to diabetic care, low public awareness, and a strong reliance on religious beliefs and traditional medicine.

Aim: This study examined the lifestyle changes of patients after lower limb amputation at Sunyani Teaching Hospital (STH) in Ghana, using the biopsychosocial model.

Methods: Semi-structured interviews were conducted on eight diabetic individuals who had undergone amputations at STH, Ghana. The study was conducted from March 2024 to May 2024.

Results: The results showed that diabetic individuals with lower limb amputation face various physical, psychological, social, and economic challenges after lower limb amputation, often depending on family support for emotional and practical help. When this support is weak, it can lead to further complications, including death.

Conclusion: The study concludes that improving the quality of life for diabetic patients with lower limb amputation in Ghana requires a comprehensive approach to post-amputation care, focusing on physical rehabilitation, emotional support, and social integration. Nurses and other healthcare professionals play a key role in this process by providing ongoing education and emotional support and working closely with patients' families and communities.

## Introduction

Diabetes mellitus is a disease that poses significant public health challenges worldwide, especially in low- and middle-income countries like Ghana [1]. Among individuals with diabetes, complications such as peripheral neuropathy and vascular disease can lead to severe outcomes, including lower extremity amputations (LEA) [2]. The management of diabetes and its complications is often complex, requiring a multidisciplinary approach that addresses not only the physical aspects of care but also the psychological, social, and economic dimensions of recovery [3]. In Ghana, the situation is exacerbated by limited healthcare infrastructure, low levels of diabetes awareness, and restricted access to rehabilitation services, leaving many patients with lower limb amputation with difficulties in adjusting to their new lifestyle [4]. Amputation refers to the surgical removal of part or all of a limb. It is typically performed to eliminate non-functional tissue, restore circulation to the remaining limb, alleviate pain, create a functional stump for prosthetic use, and ultimately improve the individual's overall well-being [5]. Lifestyle adjustment following lower limb amputation is complex, encompassing physical rehabilitation, psychological adaptation, and social reintegration. Studies show that amputees often face challenges such as chronic pain, reduced mobility, depression, social isolation, and financial constraints [6]. These challenges are more pronounced in resource-limited settings like Ghana, where access to prosthetics, physiotherapy, and psychological counselling is limited, and family members are often the primary source of support [7]. The Sustainable Development Goals (SDG), particularly Goal 3, which seeks to ensure healthy lives and promote well-being for all at all ages, underscore the importance of addressing the healthcare needs of people living with chronic diseases, including diabetes, and improving their access to quality care [8].

Furthermore, SDG 10 focuses on reducing inequalities by ensuring that all individuals, regardless of their socioeconomic status, have access to appropriate healthcare and support systems. In the context of patients with diabetic amputations, this means providing equitable access to rehabilitation services and ensuring that socioeconomic challenges do not impede their recovery and reintegration into society [9]. Addressing the lifestyle adjustment challenges of diabetic-related lower limb amputation not only aligns with these global goals but also contributes to the broader aim of achieving sustainable, inclusive development by enhancing the well-being of vulnerable populations.

This study explores the lifestyle adjustments of individual patients who have undergone LEA at Sunyani Teaching Hospital (STH) in Ghana, using a qualitative approach. It seeks to understand how these patients cope with the physical, psychological, and social aspects of their new reality, and how healthcare providers and the broader community can support their adaptation process. By addressing the gaps in care and support systems, this research aligns with the global agenda of the SDGs to improve health outcomes and reduce inequalities in healthcare access.

## Materials and methods

Study setting

The study was conducted at STH, located in the Bono Region of Ghana. The STH has a 300-bed capacity and serves as a referral centre for the Middlebelt of Ghana including the neighbouring Cote d’Ivoire. Aside from the diabetic care clinic, it offers orthopaedic, physiotherapy and palliative care.

Study design and population

An exploratory study was carried out to provide a foundation for future research, utilizing a qualitative approach to investigate the lifestyle adjustments of patients with diabetic amputations.

Patients were eligible for inclusion if they had undergone amputations at any level of the lower limbs including the foot, ankle, below the knee, knee, above the knee, and hip and had consented to take part in the study. Data collection spanned from March 2024 to May 2024.

Data collection was done after obtaining approval from the Committee for Human Research Publication and Ethics (Ref: CHRE/AP/178/023) of the University of Energy and Natural Resources and a written permit from the STH. The following ethical issues were applied throughout the study with the aim of protecting the participants: respect for human dignity, beneficence and justice. The interview guide (see Appendices A and B) was reviewed by a panel of three experts in qualitative research to ensure the appropriateness and relevance of the questions. Feedback from the piloting was used to revise and refine the questions.

Participants were selected based on specific criteria: they were aged 35 to 76 years (the typical age range for non-traumatic amputations), fluent in English, Bono, or Twi (languages understood by the investigators), and were present at the hospital during the data collection period.

Patients were excluded if they were unable to communicate in English, Bono, or Twi, were unable to give consent, had undergone traumatic amputations, had a psychotic disorder or were outside the specified age range considered in this study.

A purposive sampling technique was employed to recruit participants. Recruitment continued until data saturation was achieved with the eighth participant, at which point no new significant information emerged. By the eighth interview, the range of experiences and perceptions had become consistent, and the study's clear focus allowed for comprehensive data collection with a smaller sample size.

**Figure 1:**
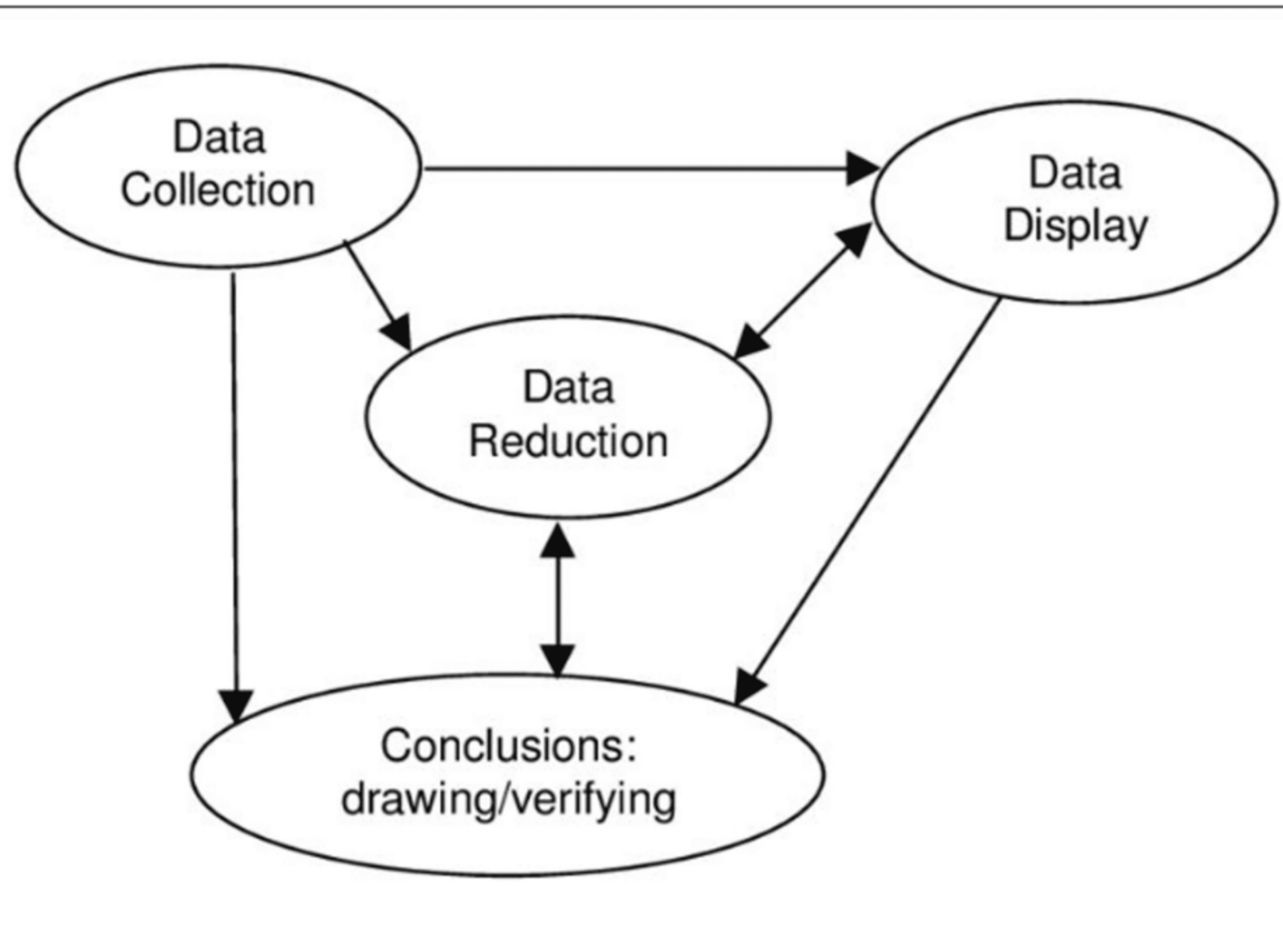
Huberman and Miles' model of qualitative data management (first uploaded by Alice Merner Agogino) Credit: Reference [10]

A semi-structured interview guide, designed specifically for this study, was used to collect data. The interview guide showed moderate to strong content validity, with a Scale Content Validity Index/Average (S-CVI/Ave) of 0.835 and Item Content Validity Index (I-CVI) values ranging from 0.67 to 1.00, according to experts. The guide consisted of eight questions based on the study's research objectives (see Figure 1). Interviews were recorded using an audio recorder (DVT4110 VoiceTracer of Phillips; Philips, Amsterdam, Netherlands) and supplemented by field notes. All interviews were conducted face-to-face at the orthopaedic unit of STH or in participants' homes, based on their convenience and preference. Each interview lasted between 30 and 45 minutes. Interviews were conducted in English, Bono, and Twi by two trained investigators with expertise in qualitative data collection and transcribed verbatim by language experts. The process involves an initial translation by a bilingual expert, back-translation by an independent expert to identify discrepancies, review by a panel of linguistic and subject matter experts for conceptual clarity and cultural appropriateness, pretesting with the target population for feedback, refinement of the translation, verification for accuracy and consistency, and thorough documentation for transparency. A pretest was conducted with three participants who shared similar characteristics with the study population. Observations from the pretest were incorporated to improve the relevance and clarity of the questions.

The recorded interviews were transcribed verbatim and categorized into themes for analysis. Transcripts were returned to participants who were willing to make more inputs and comments.

The thematic content analysis was carried out manually, following Miles and Huberman’s framework [10] of data cleansing. Miles and Huberman’s framework for data cleansing focuses on systematically refining qualitative data to ensure its clarity, relevance, and accuracy. Interview transcripts were reviewed, coded, and organized based on the biopsychosocial model to identify relevant patterns while excluding irrelevant information. Finally, conclusions were drawn and verified through triangulation, member checking, and iterative refinement to ensure credibility and minimize bias. This involves identifying and addressing inconsistencies, errors, or irrelevant details while preserving the data's original meaning. The findings were grouped into two primary categories: physical experiences and coping strategies, which were pre-determined by the structure of the interview guide.

Trustworthiness of the study

Trustworthiness was ensured using Lincoln and Guba, 1985 [11] criteria. The patients were engaged extensively to ensure that the questions posed were understood and their experiences were accurately captured. Also, a detailed description of the research context was provided to participants. The study objective and process were clearly described to the participants to gain their consent or otherwise during the data.

## Results

The data analysis revealed two main themes. To validate the qualitative findings, a literature review on the subject was conducted alongside the data analysis. Consequently, the results and discussion are presented together, incorporating relevant quotations and supporting literature to substantiate the findings.

Physical challenges

Adjustment to Mobility and the Current State of Their Body Appearance

Patients with lower limb amputation bemoaned limited ability to move saying they have to learn to walk all over again with the aid prosthetics, walking aids, or wheelchairs. They stressed that learning to use these aids can be physically taxing.

Patient 3: "Just look at me, I could go to farm freely without being dependent, now here I am I have to be aided to walk even into places where my privacy is required…"

Overcoming Pain and Funny Feelings

Patients with amputation are reported to have experienced ongoing pain or sensations from the missing limb, which affect their ability to carry out daily tasks. This pain they said could be gnawing and provocative.

Patient 1: "I fell one day when I forgot that my right leg is no more, I got up from the bed as if I was daydreaming only to tumble on the floor."

Patient 4: "Most at times I feel excruciating pain from my non-existing leg. People see this to be funny but I know what I am feeling."

Contending With Complications, Infection and Regret

Some of the patients decry complications relating to healing at the site of amputation. Many believed that after the amputation, they would be free from all complications. These usually leave most regretting after the amputation procedure.

Patient 5: "I thought having the legs cut off will have solved the whole situation but look at me with a wound so smelling and not healing. I feel less human sometimes."

Patient 6: "I have had a lot of complication aftermath, my condition I see to be worst of…though I am praying and expecting Gods’ intervention."

Changes in lifestyle

Amputation alters patient’s lifestyles and denies them certain social responsibilities and entitlements. A male who was a driver was worried about his occupation and ability to take care of the family looking at the current condition.

Patient 2: "Just look at me I have been in the hospital for almost a month now, my resources are gone, I cannot drive my car again to make to make ends meet…it seems to me…my world come crushing."

Patient 7: "I see myself facing neglect as I have lost my recognition as a title holder….I can be respected as a chief as before….it’s really hurting."

Functional loss and dependence

Being independent enables the individual to be able to perform activities of daily living with little or no assistance. The amputees said their performance has been compromised as they have to now depend much on others when performing tasks. Functional independence makes it possible to perform daily living tasks without help. One of the female amputees decries to perform a task with assistance.

Patient 8: "I have turned a baby overnight, just imagine me, I cannot cuddle my baby, cook, or bath without someone assisting me I cannot do this on my own, for how long will this be?’’

Tasks shifting and changes in family role

Participants stated that the roles that they played in the family have altered, hence, calling for compulsory task shifting.

Patient 6: "The condition that I find myself in now has rendered me unemployed and this has burdened my wife who has to add that to herself….I can see crises in within the family in time to come Hmmm."

Emotional disturbance

Losing a limb led to feelings of altered self-image, low self-esteem, and even depression, as patients struggle to accept their new physical appearance. Many amputees experience anxiety over their physical limitations and future, as well as depression due to the emotional stress of their condition. Others said the need for assistance with everyday tasks can lead to feelings of helplessness, frustration, and reduced self-worth.

Patient 5: "See me if I were married then it will have been better off but now here I am incomplete…will I get a suitor? What happens to me going forward? Only God knows…’’

Patient 3: "I feel like ending it all as visitors would come accumulating saliva in their mouth whiles communicating with me…I know that my wound smells…this makes me feel like ending it all….’’

Patient 7: "It is difficult because I have become a burden…hmm. People look after me which makes me somehow useless….”

Economic and financial constraints

Many amputees find it difficult to continue working, leading to financial hardships and dependency on family or social support systems. Also, the financial burden of medical care, including prosthetics, rehabilitation services, and medications, can be overwhelming, particularly in low-resource settings like Ghana. One echoed (Patient 2), “It has really affected me as I have spent all that I have on my condition, buying medication, walking aids and going for rehabilitation yet I have lost my job as a driver. I can no longer take care of my family as before and this is a headache."

Social challenges and discrimination

The limited mobility and dependence on others have resulted in reduced social interactions, leading to loneliness and isolation from family, friends, and community activities. Adding to it is the stigma and discrimination associated with amputation. This can hinder their social reintegration and acceptance.

Patient 7: "I feel bad to be seen as a title man and a chief of my community, I felt I should not have had the amputation but they said my life depended on it….just see me…how can I go for a community gathering on a wheel chair…"

Coping strategies

Modified Daily Activities

Some of the amputees said they have adjusted to routine activities, such as cooking, dressing, and personal hygiene, by adapting their surroundings or using assistive tools. They may restructure their living spaces to accommodate their physical limitations, making tasks easier to perform independently.

Patient 8: "Things are becoming normal for me, I have been able to adapt to certain key activities such as bathing self, eating and even exercising with the aid of my walking stick. I know that one day if I am able to get a device that will enable me to walk, I will spring back to my feet fully."

Positive Thinking and Acceptance

Most found reassurance in being alive, believing that despite their debilitating situation, life was far better than being dead and gone. They encourage themselves with some of the disabled people who have made a name despite the life challenges they face. This self-encouragement boosts their morale and helps them to cope with the predicament. This is seen in the quotation.

Patient 5: "I am alive and that is the ultimate…some were better than me yesterday but they are no more. I can do better with time and will be able to function well with aid.”

Patient 7: "I thought I had the worst of all until I saw another patient with bilateral limb amputation being cheerful…from that time on I saw that I was better."

Religious and spiritual beliefs

Many of the amputees turn to their faith for emotional resilience. They seek the face of God through prayers, spiritual counselling, and involvement in religious activities. This gives them strength and hope. Some patients trusted in the almighty God saying, "He knows whatever is good for mankind and permits all happenstance." This is reflected in these quotes:

Patient 3: "Whatever happen to us is permitted by Him (God) so I have nothing to say but to trust in Him. He knows from the beginning to the end."

Patient 8: "I will not have like this situation but here I am with it. Who am I to fight him (God). He permits whatever He wills and I will surely trust in Him."

Family and significant others' support

The findings revealed that most of the amputees were males which corresponds with several findings in West Africa. Males are usually engaged in activities that involve a higher degree of risk. Some amputees have a supportive family that assists them with day-to-day activities, supporting them to enable them to gain independence. Support they say usually comes from the immediate family members, spouse and children and significant others. These individuals provided support in the form of advice and financial assistance and helped amputees in managing the home.

Patient 4: "If they were not around me, I cannot fathom what will have happened to me. Since my admission my husband, friends and children has been very supportive…I owe them gratitude."

Patient 2: "I don’t know how it would have been…if my wife and children were not supportive, they have been around me trying to make me happy."

## Discussion

This study explored the lifestyle changes experienced by individuals with diabetes-related lower limb amputation at the STH, using the biopsychosocial model as a guiding framework. The findings provided valuable insights into the demographic, cultural, and socioeconomic factors influencing post-amputation adaptation and highlighted the critical need for tailored multidisciplinary care strategies.

The study's demographic profile showed middle-aged and older adult dominance among participants, reflecting a higher prevalence of diabetes-related complications in these age groups. This is consistent with global evidence indicating that diabetes prevalence increases with age due to cumulative risk factors such as obesity, sedentary lifestyles, and chronic stress [12]. Also, studies in Sub-Saharan Africa and Southeast Asia have similarly identified middle-aged adults as a high-risk group, emphasizing the importance of age-specific interventions [13].

The study's findings also revealed that most participants were self-employed, suggesting potential barriers to accessing workplace health benefits. This aligns with the observations of Owolabi et al. (2022) [14], who noted that informal sector workers in Ghana often face limited access to structured healthcare services, exacerbating their vulnerability to chronic disease complications. Again, financial barriers were a recurring theme, as participants reported struggling to afford prosthetics and rehabilitation services. These challenges mirror findings in Nigeria, where limited healthcare infrastructure and high costs impede access to comprehensive post-amputation care [15].

Participants reported experiencing significant emotional and psychological burdens, including anxiety, depression, and altered self-image, which is consistent with earlier studies. A study in India reported that amputees frequently grapple with heightened emotional distress and social stigma, often compounded by inadequate psychological counselling [16], which is scarce to access. Similarly, findings in Ghana by Morgado Ramirez et al. (2022) [17] revealed that amputation often triggers profound social isolation, especially in contexts where physical disability is culturally stigmatized.

The limited availability of rehabilitation services, including prosthetics and physiotherapy, was a significant barrier for participants. This is consistent with findings from South Africa and Bangladesh, where financial constraints and inadequate healthcare infrastructure leave many amputees reliant on family members for mobility and basic daily tasks [18,19]. In contrast, developed countries like Canada and the United States reported higher rehabilitation success rates due to better healthcare resources, underscoring the disparities in global healthcare access [20].

Religious faith emerged as a critical coping strategy for participants, aligning with observations in other regions. For instance, a study in North America found that spiritual beliefs provided patients with amputation with emotional resilience, enabling them to accept their circumstances and adapt to their new reality [21]. In Ghana, participants relied heavily on family and religious communities for both emotional and financial support, echoing findings by Boamah and Druye (2023) [22], who emphasized the role of informal support systems in managing chronic illness including patients with diabetes-related amputation.

The economic impact of amputation, including loss of employment and high treatment costs, was a major challenge for participants. Similar patterns have been reported in Cameroon, where amputated patients face significant difficulties reintegrating into society due to financial instability and limited employment opportunities [23]. The loss of independence and reliance on informal networks further underscores the urgent need for socioeconomic support interventions.

Limitations

The research was limited to a single public orthopaedic facility, restricting the generalizability of the findings to other hospitals. Additionally, the inclusion of a broader population or multiple facilities could have provided more diverse perspectives and yielded key information that may have been overlooked, limiting the comprehensiveness of the study.

## Conclusions

Patients with diabetic-related amputation faced myriad challenges that cut across biopsychosocial domains. Lack of access to healthcare, financial hardships, and limited rehabilitation services aggravate the existing challenges. Familial support and religious beliefs served as vital coping mechanisms offering some comfort and resilience to diabetic individuals. A comprehensive approach that requires timely counselling and provision of sustainable income sources brings respite to individuals with amputation and improves their quality of life. This study serves as a basis for further work.

## Figures and Tables

**Figure 2:**
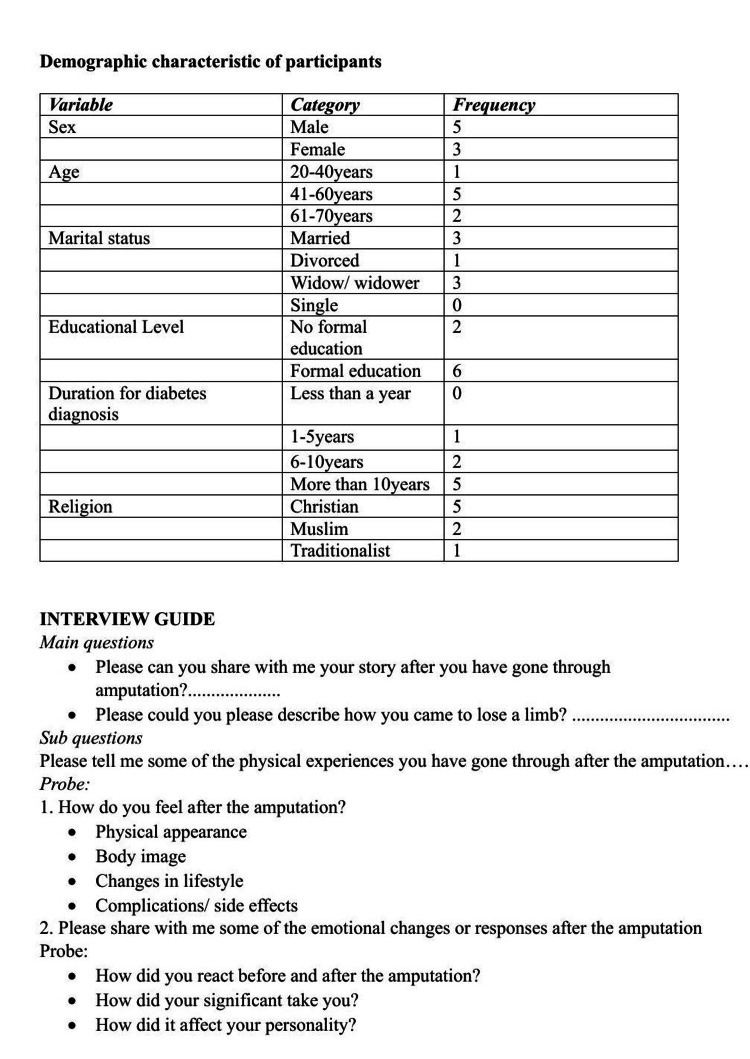
Table and interview guide

**Figure 3:**
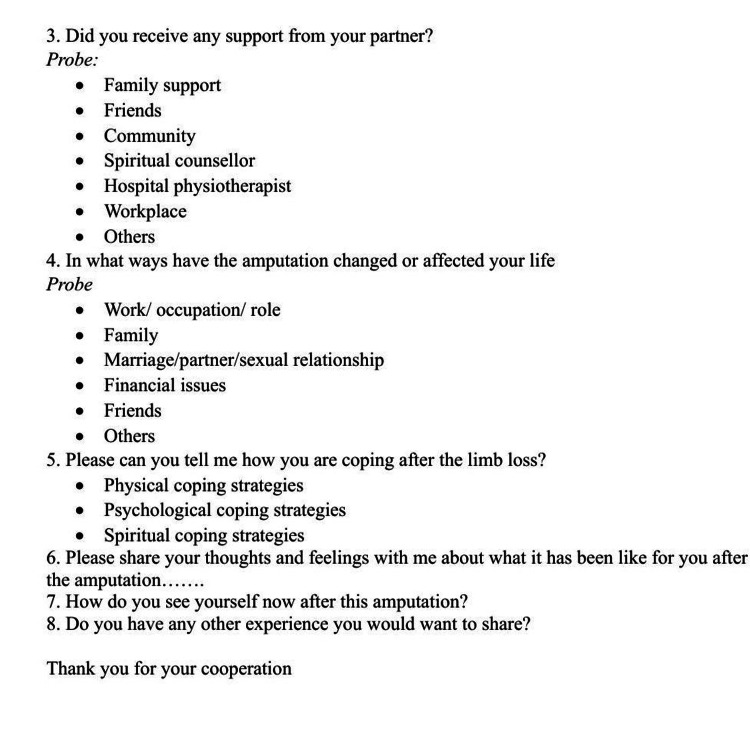
Table and interview guide

## Appendices

Appendix A 

Appendix B 
